# Germline Genetic *NBN* Variation and Predisposition to B-cell Acute Lymphoblastic Leukemia in Children

**DOI:** 10.21203/rs.3.rs-3171814/v1

**Published:** 2023-07-21

**Authors:** Carolin Escherich, Wenan Chen, Yizhen Li, Wenjian Yang, Rina Nishii, Zhenhua Li, Elizabeth A. Raetz, Meenakshi Devidas, Gang Wu, Kim E. Nichols, Hiroto Inaba, Ching-Hon Pui, Sima Jeha, Bruce M. Camitta, Eric Larsen, Stephen P. Hunger, Mignon L. Loh, Jun J. Yang

**Affiliations:** 1 Department of Pharmacy and Pharmaceutical Sciences, St. Jude Children’s Research Hospital, Memphis, TN, USA; 2 Department for Pediatric Oncology, Hematology and Clinical Immunology, Medical Faculty, Heinrich-Heine University, Duesseldorf, Germany; 3 Center for Applied Bioinformatics, St. Jude Children’s Research Hospital, Memphis, TN, USA; 4 Department of Pediatrics and Perlmutter Cancer Center, New York University Langone Health, New York, NY, USA; 5 Department of Global Pediatric Medicine, St. Jude Children’s Research Hospital, Memphis, TN, USA; 6 Department of Oncology, St. Jude Children’s Research Hospital, Memphis, TN, USA; 7 Department of Pediatrics, Midwest Center for Cancer and Blood Disorders, Medical College of Wisconsin, Milwaukee, WI, USA; 8 Department of Pediatrics, Maine Children’s Cancer Program, Scarborough, ME, USA; 9 Department of Pediatrics and Center for Childhood Cancer Research, Children’s Hospital of Philadelphia and Perelman School of Medicine, University of Pennsylvania, Philadelphia, PA, USA; 10 Seattle Children’s Hospital, the Ben Towne Center for Childhood Cancer Research, University of Washington, Seattle, WA, USA

## Abstract

Biallelic mutation in the DNA-damage repair gene *NBN* is the genetic cause of Nijmegen Breakage Syndrome, which is associated with predisposition to lymphoid malignancies. Heterozygous carriers of germline *NBN* variants may also be at risk for leukemia development, although this is much less characterized. We systematically examined the frequency of germline *NBN* variants in pediatric B-ALL and identified 25 putatively damaging *NBN* coding variants in 50 of 4,183 B-ALL patients. Compared with the frequency of *NBN* variants in 118,479 gnomAD non-cancer controls we found significant overrepresentation in pediatric B-ALL (*p*=0.004, OR=1.77). Most B-ALL-risk variants were missense and cluster within the NBN N-terminal domains. Using two functional assays, we verified 14 of 25 variants with severe loss-of-function phenotypes and thus classified these as pathogenic or likely pathogenic. Finally, we found that heterozygous germline *NBN* variant carriers showed similar survival outcomes relative to those with WT status. Taken together, our findings provide novel insights into the genetic predisposition to B-ALL, the impact of *NBN* variants on protein function and suggest that heterozygous *NBN* variant carriers may safely receive B-ALL therapy.

## Introduction

B-cell acute lymphoblastic leukemia (B-ALL) is the most common cancer in children, and there is growing evidence for the inherited basis of ALL susceptibility.^1^ The majority of leukemia risk genes identified thus far are involved in either lymphoid differentiation (*e.g., ETV6*, *PAX5*, *IKZF1*, and *TCF3*)^1–5^ or cell cycle and apoptosis signaling (*e.g.*, *CDKN2A* and *TP53*).^6,7^ DNA damage repair has also been implicated in the pathogenesis of both lymphoid and myeloid leukemias. Germline pathogenic variants in DNA repair genes interfere with the correction of DNA double-strand breaks (Ataxia Telangiectasia^1^ and Nijmegen Breakage Syndrome^8^), single-strand breaks (Constitutional Mismatch Repair Deficiency)^9^, or inter-strand crosslinks (Fanconi Anemia).^10^ In these conditions, the failure of DNA repair leads to genome instability and thus increased risk of hematological disorders.^1^

Nijmegen Breakage Syndrome (NBS) is an autosomal-recessive condition, caused by a biallelic loss-of-function (LOF) mutation in the *NBN* gene.^8^ About 90% of NBS patients are homozygous for the frameshift mutation, p.K219fs (c.657_661delACAAA), which is noted to be a founder mutation in populations of Eastern European (Slavic) descent.^11,12^ The NBN protein functions as a sensor for DNA double-strand breaks and an adaptor for the downstream repair signaling.^11^ The N-terminus contains a Forkhead-Associated and two Breast Cancer C-terminus domains (FHA-BCRT-repeat domain). This region allows interaction with the Mediator of DNA Damage Checkpoint 1 (MDC1) and phosphorylated Histone H_2_AX, both of which accumulate at the site of DNA damage.^13,14^ The NBN C-terminus harbors the MRE11/RAD50 and ATM interaction sites, and thus implicated in cell cycle control, DNA repair, and apoptosis signaling.^15,16^ NBS patients develop characteristic phenotypes including immunodeficiency, radiosensitivity, and cancer susceptibility.^8,17^ The cumulative risk of developing cancer during childhood among NBS patients is up to 70%, and lymphoid malignancies (B- or T-linage subtypes) are by far most frequently diagnosed.^18,19^

Heterozygous carriers of pathogenic *NBN* variants are clinically asymptomatic but are still believed to be at an increased risk for cancer development.^20^ Two large studies (with >34·000^21^ and >39·000^22^ cancer patients) confirmed pan-cancer association with heterozygous *NBN* p.K219fs variant carriers, who are particularly prone to the development of breast cancer, prostate cancer, leukemia and lymphoma.^23,24^ Besides truncating *NBN* variants, more than 160 missense germline *NBN* variants have been identified,^21^ including the *NBN* variant p.I171V noted in pediatric B-ALL.^25–27^ Other germline *NBN* variants (*e.g.*, p.S93L, p.D95N, p.V210F and p.R215W) were described in patients with B-ALL, but with conflicting evidence regarding their impact on leukemia risk.^23,25,26,28^

Although there are a growing number of *NBN* variants detected in cancer patients, efforts to assess their association with B-ALL predisposition are limited. Also, the functional consequences of these variants remain largely uncharacterized, thus hampering clinical interpretation of variant pathogenicity. To address these challenges, we comprehensively screened for germline *NBN* variants in a large cohort of 4,183 pediatric B-ALL patients, experimentally characterized these variants using two phenotyping assays, and evaluated their association with B-ALL characteristics and treatment outcomes.

## Results

### Targeted sequencing of the *NBN* gene in pediatric B-ALL.

**Figure 1.**
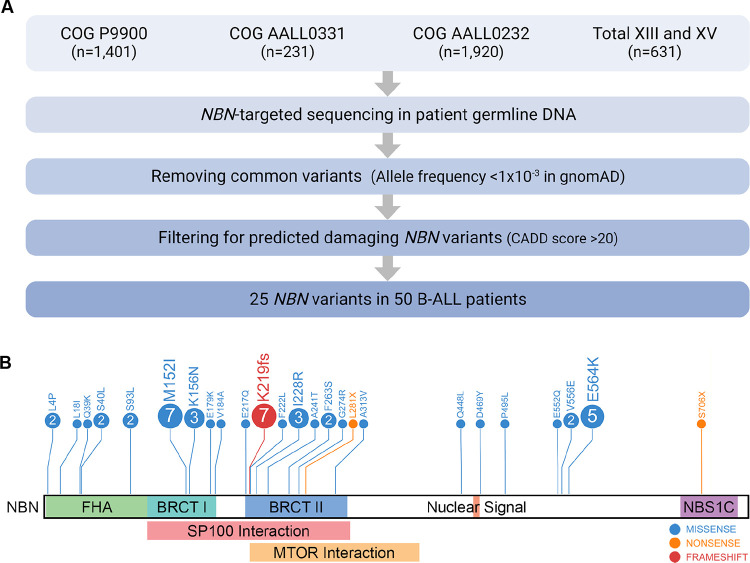
Workflow for *NBN*-targeted sequencing in pediatric B-ALL patients. **A)** CONSORT diagram of COG and St. Jude patients included in this study. **B)** Protein domain plot of NBN (NM_002485): Forkhead-associated Domain (FHA), Breast Cancer C-terminal domain (BRCT) I and II, MRE11 and ATM interaction site (NBS1C), SP100 interaction site, and MTOR interaction site. The upper panel shows the amino acid substitutions predicted to result from the germline *NBN* variants identified in this study. The numbers in circles indicate the number of patients that harbor the *NBN* variant of interest.

To comprehensively characterize the pattern and prevalence of germline *NBN* variants in pediatric B-ALL, we performed targeted sequencing of all exons of the *NBN* gene in 4,183 children with newly diagnosed disease enrolled on three COG and two St. Jude frontline clinical trials (Figure 1A). Putative damaging *NBN* variants were identified based on two criteria: (1) a population allele frequency <1×10^−3^ in the general population derived from the gnomAD v2.1 dataset,^29^ and (2) a Combined Annotation Dependent Depletion (CADD) Score >20.^30^ The allele fraction of each variant in each sample was confirmed to be approximately 50%, consistent with a heterozygous genotype in all *NBN* variant carriers.

Overall, we discovered 25 putative damaging *NBN* coding variants in 50 B-ALL patients, representing a cumulative incidence of 1.2% (Figure 1B and Table 1), and we considered these as potentially related to ALL risk. Compared with only 208 *NBN* variants found in 208 of 118,479 non-cancer individuals in the gnomAD v.2.1 Exomes cohort (0.2%),^29^ putative damaging *NBN* variants were significantly overrepresented in B-ALL patients (*p*=0.004, OR=1.77; **Supplemental Figure 1**). Notably, four of 25 B-ALL-related *NBN* variants were not reported in the gnomAD database (Table 1). Three of the B-ALL-related *NBN* variants found in nine patients resulted in protein truncation, including the known loss-of-function variant p.K219fs (Figure 1B and Table 1).^11^ The remaining 22 variants were missense and preferentially located in the N-terminal FHA-BRCT-repeat domain (16 of 22 variants, 72.7%; Figure 1B).

### Functional characterization of *NBN* variants.

**Figure 2.**
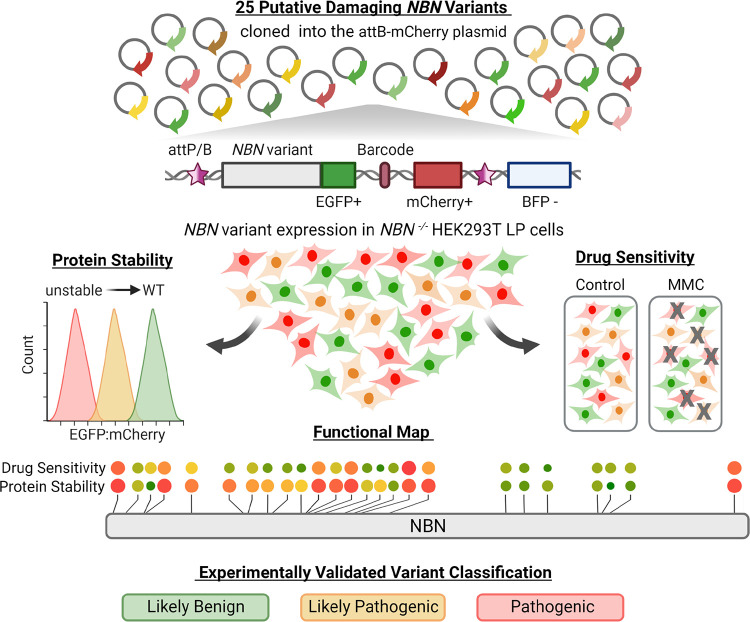
Experimental design for *NBN* variant functional characterization. Parallel *NBN* variant characterization was done using the engineered *NBN*^−/−^ HEK293T LP cell line model. First, the 25 variants of interest were fused to EGPF, tagged with a unique barcode sequence, and cloned in the attB-mCherry recombination plasmid. Next, *NBN* variants were introduced into the attP/attB recombination site in *NBN*^−/−^ HEK293T LP cells. Cells with successful recombination were identified as mCherry^+^/BFP^−^ population in flow cytometry. Finally, *NBN* variant expressing cells were subjected to two different types of phenotyping to determine their effect on 1) NBN variant protein stability or 2) NBN variant mitomycin C (MMC) sensitivity *in vitro*. NBN protein stability was quantified by the fluorescence intensity of the EGFP fusion protein and normalized to the co-translationally expressed mCherry fluorescence signal (EGFP:mCherry ratio). Unstable variants resulted in decreased EGFP expression and thus a low EGFP:mCherry ratio, as illustrated by the red-colored histogram. Drug sensitivity was determined by the change in *NBN* variant frequency after MMC exposure. Damaging variants resulted in the loss of NBN signaling during MMC-induced DNA damage repair. This led to reduced cell survival and thus under-representation of the respective variants after MMC treatment, which was quantified by targeted sequencing of the barcode region. Finally, NBN variant protein stability and MMC drug sensitivity were both considered for *NBN* variant classification.

Of the 25 B-ALL-related *NBN* variants in our cohort, only four variants (16%) are classified as benign or pathogenic as reported in the ClinVar database, with the remaining 21 variants (84%) noted as “of uncertain significance” (Table 1). To comprehensively characterize B-ALL-related germline *NBN* variants, we utilized the HEK293T Landing Pad model to examine variant function at a single cell level.^31,32^ First, we knocked out the endogenous *NBN* gene by CRISPR/Cas9 editing (hereafter, *NBN*^−/−^ HEK293T LP cells). Next, we generated the 25 *NBN* variants of interest by site-directed mutagenesis, with each variant tagged with an EGFP-fusion protein and a unique barcode index.^33^ *NBN* variants were introduced into the AAVS safe harbor locus (attP site) of *NBN*^−/−^ HEK293T LP cells by homology recombination such that each cell expresses only a single variant of interest. Finally, *NBN* variants were subjected to two phenotyping assays to determine their effect on: 1) NBN protein stability, and 2) mitomycin C (MMC) drug sensitivity *in vitro* (Figure 2).

**Figure 3.**
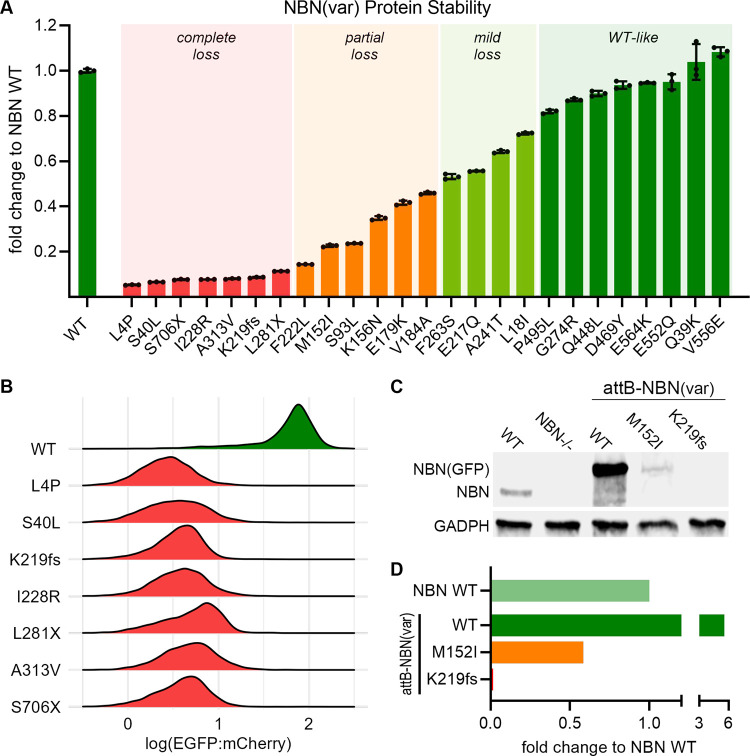
NBN variant protein stability screen. **A)** Presentation of the fold change of NBN variant EGFP:mCherry ratio compared with WT NBN as quantified by flow cytometry: Complete loss <0.1, Partial loss 0.1–0.5, Mild loss 0.5–0.8, WT-like >0.8. The results are presented as the mean of three independent experiments +/− standard deviation. **B)** The pattern of EGFP:mCherry distribution in unstable NBN variants (red) compared with WT NBN (green). Each histogram was generated from ~4,000 NBN (WT or variant) expressing cells. **C)** Western blot analysis of NBN (WT or variant) protein expression level. Lanes 1–2 show the absence of WT NBN protein expression in *NBN*^−/−^ HEK293T LP cells (lane 2) compared to WT HEK293T LP cells (lane 1). Lanes 3–5 depict NBN (WT or variant) protein levels after re-expression in *NBN*^−/−^ HEK293T LP cells. Successfully recombined cells were identified as mCherry^+^/BFP^−^ population and separated by flow cytometry prior to protein extraction and Western blot analysis. The EGFP fusion protein resulted in a slight increase in the molecular weight. **D)** Relative NBN variant expression level compared with WT NBN expression as quantified using Western blot.

We focused on protein stability because this is a known determinant of NBN activity. Also, in NBS patients with p.K219fs genotype, the expression level of the alternatively translated protein, p70, significantly correlates with cancer risk.^34^ We determined protein stability for each *NBN* variant by measuring the fluorescence intensity of the EGFP fusion protein as a proxy marker for the variant protein abundance (Figure 2).^31,33^ As shown in Figures 3A and B, wild-type NBN tagged with EGFP resulted in robust green fluorescence as measured with flow cytometry. By contrast, the loss of function *NBN* variant, p.K219fs, led to an approximately 10-fold reduction in the EGFP signal. Applying this assay to all B-ALL-related *NBN* variants, we identified another six variants that resulted in unstable protein, defined as ≥ 90% reduction of EGFP intensity compared to WT NBN-EGFP (Figure 3A). As expected, all three of the truncating variants resulted in loss of protein expression. However, four of the unstable variants are missense, leading to single amino acid substitutions at positions 4, 40, 228, and 313 and resulting in complete loss of protein stability as well (Figures 3A and B). Six missense variants showed partial loss of NBN protein stability (defined as 50–90% reduction of EGFP intensity); four variants showed only mild loss (20–50% reduction of EGFP intensity); and eight variants had WT-like protein stability (<20% reduction of EGFP intensity). To validate these results using orthogonal assays, we selected the most common missense variant, p.M152I, and the most common truncating variant, p.K219fs, to perform Western blot analysis (Figures 3C and D).

**Figure 4.**
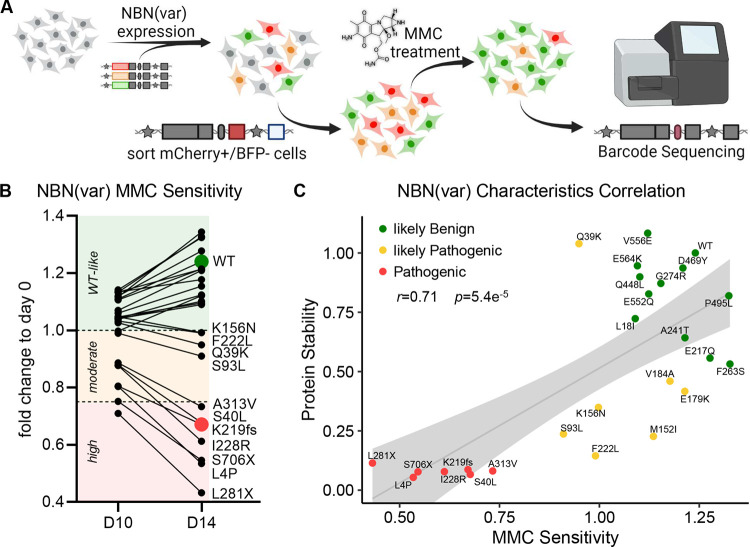
*NBN* variant mitomycin C drug sensitivity screen. **A)** WT *NBN* or 25 *NBN* variants were simultaneously expressed in *NBN*^−/−^ HEK293T LP cells. Successfully recombined cells were identified as mCherry^+^/BFP^−^ population, separated by flow cytometry, and cultured in media supplemented with MMC 20nM for 10 or 14 days. *NBN*^*−*/−^HEK293T LP cells expressing WT NBN or WT-like variants (green) became more tolerant to MMC-induced DNA damage, which resulted in higher proliferation compared to cells expressing NBN variants with reduced (orange) or loss of function (red) activity. MMC drug sensitivity was determined by the fold change in variant frequency before and after MMC exposure and was quantified by Illumina MiSeq of the barcode sequence for each variant. **B)** *NBN* variant abundance during 20nM MMC treatment for 10 and 14 days was measured as the fold change to day 0 of the normalized barcode reads. MMC sensitivity was classified as high (<0.75), moderate (0.75–1) and WT-like (>1). Each dot represents an average fold change of nine measurements, which derive from three barcodes assigned to each *NBN* variant and each condition performed as triplicates. **C)** NBN variant protein stability was plotted against NBN variant MMC sensitivity. *P*-values were estimated using the Pearson correlation test (*r*).

Because protein instability is not the only mechanism for loss of activity, we sought to evaluate a cellular endpoint that more broadly reflects NBN activity. Cells derived from NBS patients (e.g., fibroblasts or lymphoblastoid cells) show increased chromosomal aberrations and decreased proliferation after DNA damage induced by irradiation or exposure to radiomimetic drugs such as MMC.^8,35,36^ In fact, MMC sensitivity testing is an established clinical assay for the diagnosis of NBS. To demonstrate the validity of this assay, we first confirmed that *NBN*^*−/−*^ HEK293T LP cells are highly sensitive the MMC and re-expression of WT NBN greatly enhanced tolerance to MMC-induced apoptosis **(Supplemental Figures 2A and B)**. By contrast, re-expression of the known pathogenic variant, p.K219fs, conferred no survival benefit during MMC treatment. Next, WT *NBN* and 25 *NBN* variants of interest were pooled into a library and were simultaneously expressed in *NBN*^*−/−*^ HEK293T LP cells. Because cells expressing a loss-of-function *NBN* variant are more susceptible to MMC-induced DNA damage, these cells are expected to undergo apoptosis quickly and the respective variants should become underrepresented after MMC exposure (Figure 4A). Based on the fold change in variant frequency at day 14 relative to pre MMC treatment, we assigned each variant as “highly sensitive” (fold change <0.75), “moderately sensitive” (fold change 0.75–1) or “WT-like” (fold change >1), respectively (Figure 4B). Besides the known loss-of-function variant, p.K219fs, two truncating variants (p.L281X and p.S706X) and four missense variants (p.L4P, p.S40L, p.I228R, and p.A313V) showed poor survival during MMC treatment, and were thus designated as highly sensitive. In addition, four variants (p.Q39K, p.S93L, p.K156N, and p.F222L) were tested as moderately sensitive to MMC.

### *NBN* variant classification based on protein stability and mitomycin C sensitivity.

Based on functional characterization results, we classified the 25 B-ALL-related *NBN* variants as “pathogenic”, “likely pathogenic” and “likely benign”, as summarized in Table 2. Comparing the results from both screening approaches, we found a strong correlation between *NBN* variant protein stability and MMC drug sensitivity (*r*=0.71, *p*=5.4×10^−5^, Pearson correlation test, Figure 4C). Besides p.K219fs, six variants were linked to both unstable protein and high sensitivity to MMC, and thus designated as pathogenic (Figure 4C, Table 2). Further, 11 variants were found to have WT-like MMC tolerance and WT-like or mild loss of NBN protein stability and were therefore assigned as likely benign. Finally, seven variants were considered as likely pathogenic due to partial loss of protein stability and/or moderate sensitivity to MMC.

### Pathogenic *NBN* variants in B-ALL cases cluster in the N-terminal functional domains.

**Figure 5.**
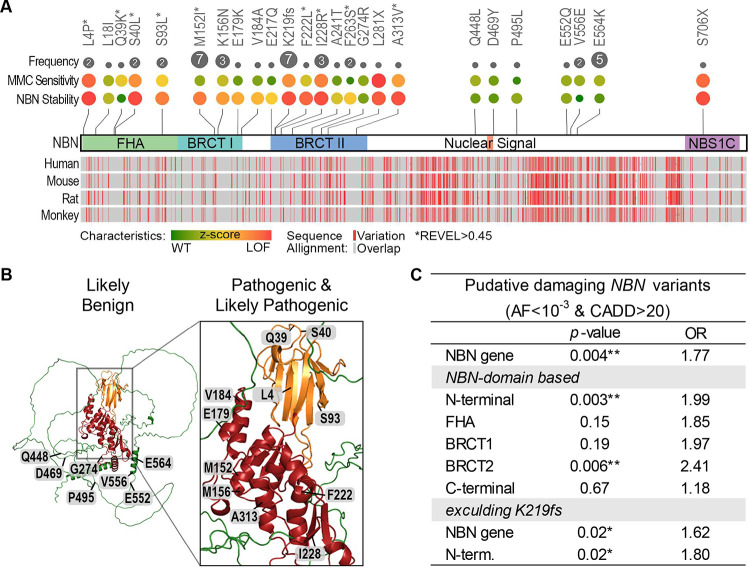
Key functional domains of NBN are preferentially affected by genetic variation. **A)** The top panel summarizes the frequency in B-ALL cases, effects on MMC drug sensitivity, and NBN protein stability for each *NBN* variant. The bottom panel depicts the alignment of NBN protein sequences from human (Homo Sapiens; NP_002476.2), mouse (Mus musculus, NP_038780.3), rat (Rattus norvegicus; NP_620228.1), and monkey (Macaca mulatta; NP_001252668.1). Protein sequence alignment was done in COBALT NCBI Multiple Sequence Alignment Viewer, Version 1.22.0. **B)** AlphaFold structure prediction of NIBRIN (AF-O60934-F1): FAH domain in orange, BRCT I & II in red, and C-terminal domain in green. Experimentally validated likely benign *NBN* variants relate to the peripheral moieties, while pathogenic and likely pathogenic *NBN* variants relate to the NBN central region. **C)** Cumulative burden of putative damaging *NBN* variants in B-ALL cases vs. gnomAD non-cancer controls calculated by ethnicity-stratified Cochran-Mantel-Haenszel test. The statistical analysis was performed for *NBN* gene-based or *NBN* domain-based rare variant burden test (*p<0.05, **p<0.001).

Of the 14 *NBN* variants experimentally validated as pathogenic or likely pathogenic (P/LP) (Table 2), 13 affect the N-terminal FHA-BRCT-repeat domain (Figure 5A). Sequence alignment confirmed this region to be highly conserved among different species and AlphaFold prediction identified the FHA-BRCT-repeat domain as a complex convoluted tertiary structure (Figure 5B).^37^ Therefore, we reasoned that sequence variation of the FHA-BRCT-repeat domain may have strong effects on NBN function. In fact, variants within the N-terminus domain showed a heightened association with B-ALL risk (*p*=0.003, OR=1.99) and remained significant even after excluding p.K219fs from the analysis (*p*=0.02, OR=1.8; Figure 5C).

### Patient characteristics and outcome analysis for germline *NBN* variant carriers.

**Figure 6.**
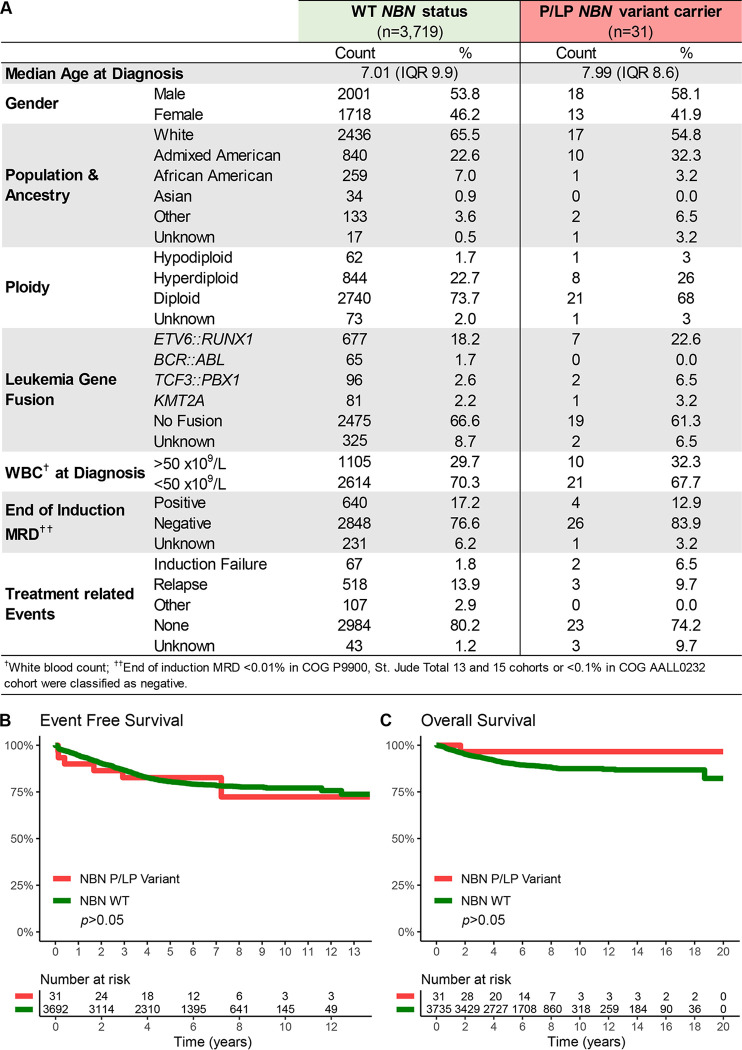
Association of *NBN* variants with clinical characteristics of B-ALL. **A)** Characteristics of B-ALL patients with experimentally validated P/LP germline *NBN* variant (n=31) were compared to those with confirmed WT *NBN* status (N=3,719) treated in COG P9900, AALL0232, St. Jude Total 13 and St. Jude Total 15 clinical trials. **B)** Event-Free Survival and **C)** Overall Survival in carriers of experimentally validated P/LP *NBN* variants and patients with WT *NBN* status.

Next, we examined the relationship between germline *NBN* status and clinical features of B-ALL (Figure 6A). Comparison of the 47 carriers of putative damaging *NBN* variants and 3,719 patients with WT *NBN* status did not reveal a significant difference in patient characteristics (age at diagnosis, sex, or genetic ancestry distribution) or leukemia genetic subtype (ploidy and fusion genes). Also, restricting the analysis to patients with experimentally validated P/LP *NBN* variants (n=31) did not show any significant association either (Figure 6A). The NBS founder mutation, p.K219fs, is most frequently found in the Eastern European population.^12,38^ However, no overrepresentation of patients with European descent was seen. In fact, B-ALL-related *NBN* variants were also frequently found in the Admixed American population.

Finally, we evaluated the association of germline *NBN* status and treatment outcomes of patients treated on two COG and two St. Jude frontline B-ALL clinical trials (AALL0232, COGP9900, St. Jude Total 13 and 15). Carriers of P/LP *NBN* variants showed similar response to induction therapy compared to those with WT *NBN* status, as measured by the minimal residual disease (MRD) (Figure 6A). Unlike NBS patients, who have been reported to have an inferior prognosis,^39^ there were also no significant differences in overall survival or event-free survival between heterozygous carriers of P/LP *NBN* variants and those with WT *NBN* status in our cohort (Figures 6B and C). Notably, no P/LP *NBN* variant carrier was diagnosed with a second malignancy during a median follow-up period of 6.15 years (Rage: 1.1 month to 13.7 years).

## Discussion

Even though carriers of a heterozygous germline *NBN* variant have been linked to cancer risk,^20,22,23,40,41^ malignancies of lymphatic origin, in particular B-ALL, were underrepresented in these studies.^21^ Therefore, evaluation of this patient group has been limited. Herein, we sought to fill the knowledge gap by screening a large cohort of pediatric B-ALL patients for germline genetic variants in the *NBN* gene. We identified significant overrepresentation of putative damaging *NBN* variants, most of which are missense. We systematically characterized the functional consequences of these variants, identifying 14 as pathogenic or likely pathogenic. Outcome analyses in four frontline ALL clinical trials at COG and St. Jude suggest that heterozygous carriers of P/LP *NBN* variants fare as well as those with WT *NBN* status. Together, our study advanced the understanding of the role of germline *NBN* variants in pediatric B-ALL predisposition and treatment.

*NBN* variants identified in targeted sequencing were prioritized based on a CADD score >20, which indicates these variants are predicted to be among the top 1% most damaging variants.^42^ However, experimental validation revealed 11 of these predicted deleterious variants as likely benign. Instead, filtering these variants based on the REVEL (Rare Exome Variant Ensemble Learner) score >0.45 prioritized nine missense variants, eight of which were experimentally validated as P/LP. Therefore, we reason that REVEL scoring provides more reliable prediction of variant pathogenicity.

The majority of known pathogenic *NBN* variants lead to protein truncation, resulting in partially functional protein (e.g., p70 or p45).^21,43^ These hypomorphic variants retain some activity in DNA damage response, and variability in their expression level seems to be linked to the degree of genome instability and cancer risk.^34^ This observation is supported by our finding that NBN protein stability and MMC drug sensitivity are strongly correlated. Besides the truncating variant p.K219fs, we describe four missense variants with complete loss of NBN protein stability. However, absence of NBN expression is embryonically lethal,^44^ such that these variants usually only occur as a heterozygous genotype.

In addition, missense *NBN* variants may severely impair NBN signaling, as has been suggested in a single NBS patient with compound heterozygous genotype for p.K219fs and p.R215W. The missense variant was characterized as pathogenic and, in conjunction with p.K219fs, resulted in a particularly severe NBS phenotype.^45,46^ In B-ALL patients, we detected pathogenic missense variants far more frequently than truncating variants, and we found missense variants to be enriched in the FHA-BRCT-repeat domain. The pathogenicity of these variants may be explained by the moderate to severe reduction in protein stability. However, these variants within the highly conserved FHA-BRCT-repeat domain may impair its ability to interact with other phosphoproteins and to recruit the MRE11-RAD50-NBS1 (MRN) complex.^13,14,47^ Consistent with this hypothesis, one likely pathogenic missense variant, p.Q39K, did not affect protein stability. This variant alters the FHA phosphoprotein-binding pocket that interacts with the endonuclease CtIP.^47^ Sequence variation at adjacent positions were shown to sensitize to irradiation or camptothecin *in vitro* but without affecting NBN stability, similar to what we observe for the NBN variant p.Q39K.^47^

NBN-deficient cells display elevated levels of baseline DNA damage, chromosomal instability, and aberrant cell cycle control,^48^ which is considered to drive cancer development. In the lymphatic tissues, loss of NBN expression results in profound defects in the hematopoietic stem-cell (HSC) and lymphoid differentiation process: (1) NBN deficient HSC show reduced self-renewal and differentiation capacities,^48^ (2) NBN deficient mice display severely impaired hematopoiesis,^48,49^ and (3) NBS patients develop a stage-specific B-cell differentiation arrest accompanied by aberrant composition of the mature B-cell compartment.^49–51^ This phenotype is attributed to the impaired resolution of Recombination Activating Gene (RAG) induced DNA double-strand breaks during V(D)J recombination,^50^ which may also explain the particularly high risk for leukemia and lymphoma development. Regrettably, we lack detailed immunophenotyping data to inform the lymphoid compartment in germline *NBN* variant carriers in our cohort, and the molecular mechanism of B-ALL development in these individuals remains to be investigated.

Family history was available based on the patient interview for six cases enrolled on St. Jude clinical trials, of whom five have an unremarkable history. Only one case, carrier of the LP *NBN* variant p.M152I and with low-hypodiploid B-ALL, showed multiple occurrences of cancer in the family history. However, this individual also has a germline variant in the *TP53* gene (rs1042522), which is well recognized to be associated with low-hypodiploid ALL.^52^ Therefore, it is unclear to what degree the *NBN* variant contributed to the familial tumorigenesis in this patient. In NBN deficient mice with T-cell Lymphoma, tumor genomic analysis revealed a characteristic mutational pattern.^51^ For two patients from the St. Jude Total 15 cohort, tumor whole-exome sequencing data was available and we performed mutation signature analyses, which was found to be unremarkable (data not shown; COSMIC Mutational Signatures, Version 3.3). These findings suggest the need for future studies exploring the impact of heterozygous *NBN* variants on lymphoid differentiation and leukemia development.

Taken together, our results highlight the importance of germline pathogenic *NBN* variants to B-ALL predisposition, which may inform clinical strategies and cancer surveillance in these children in the future.

## Materials and Methods

### Patient Cohort –

4,183 patients enrolled in Children’s Oncology Group (COG) P9900, AALL0232, AALL0331 and St. Jude Total Therapy 13 and 15 clinical trials for newly diagnosed B-ALL were included for *NBN*-targeted sequencing.^53–57^ The study was approved by institutional review boards at St. Jude Children’s Research Hospital and COG member institutions and informed consent was obtained from parents, guardians, or patients, as appropriate.

***NBN*-Targeted Sequencing –** was performed following procedures described previously.^2,7,58^ Briefly, Illumina dual-indexed libraries were generated from patient germline DNA and pooled in sets of 96 before hybridization with customized Roche NimbleGene SeqCap EZ probes (Roche NimbleGen, WI, USA) to capture *NBN* genomic region. Quantitative PCR was used to define the appropriate capture product titer necessary to efficiently populate an Illumina HiSeq 2000 flow cell for paired-end 2×100 bp sequencing.

### *NBN*^*−/−*^ HEK293T Landing Pad Cellular Model and *NBN* variant characterization –

*NBN* gene knockout single clone was generated from HEK293T Landing Pad cells^31–33^ transiently transduced with Cas9 and sgRNA targeting *NBN* (*NBN*^−/−^ HEK293T LP cells). Details of sgRNA and genotyping primers can be found in **Supplemental Table 1**. Next, *NBN* variants were integrated into the attP locus of *NBN*^−/−^ HEK293T LP cells via Bxb1-mediated recombination.^31,33^ Cells with successful recombination were identified by flow cytometry as mCherry-positive, BFP-negative (mCherry^+^BFP^−^) population. Subsequently, successfully recombined cells were used for *NBN* variant characterization.

NBN variant protein stability was tested after each variant was separately expressed in *NBN*^−/−^ HEK293T LP cells. The fluorescence intensity of the NBN-EGFP fusion protein was measured by flow cytometry and normalized to the co-translationally expressed mCherry fluorescence signal (EGFP:mCherry ratio). Each variant was tested in triplicates and protein stability was determined as the fold change to WT NBN-EGFP expression. For Western blot analysis the mCherry^+^BFP^−^ population was sorted by flow cytometry. Protein samples were separated on a Mini-PROTEAN^®^TGX^™^ Precast Gel 4–15% (Bio-Rad Laboratories, CA, USA) and stained with rabbit anti-NBS1 (NB100–143, Novus Biologicals, CO, USA) and rabbit anti-GADPH (D16H11, CST, MA, USA). IRDye^®^ 800CW Goat anti-Rabbit (Li-cor, NE, USA) was used as the secondary antibody.

For the MMC drug sensitivity assay, WT *NBN* and *NBN* variants were pooled together into a library. Next, the library was expressed in *NBN*^−/−^ HEK293T LP cells and the cell pool treated with 20nM MMC (Cat. No. S8146, Selleckchem, TX, USA) for 10 or 14 days. Cells were harvested for genomic DNA extraction, and Illumina MiSeq of the barcode region was performed to quantify *NBN* variant frequency **(Supplemental Table 4)**. Variant-barcode counts were normalized to the total barcode reads and the fold change in variant frequency after treatment compared to day 0 was used as the indicator for variant drug sensitivity. Each variant was represented by three barcodes and each MMC treatment condition was performed in triplicates.

### Statistical Analyses –

The enrichment of rare and predicted deleterious *NBN* variants in B-ALL patients was assessed using the CoCoRV pipeline,^59^ where gnomAD v.2.1 exome-based non-cancer summary counts (n=118,479) were used as the non-ALL control cohort. Putative deleterious *NBN* variants were defined as follows: allele frequency <1×10^−3^ after combining both cases and controls, protein-truncating variants (frameshift and nonsense) or missense variants with a CADD score >20. Ethnicity stratified analysis using the Cochran–Mantel–Haenszel (CMH)-exact test was used to calculate the *p*-values. Both gene-based and specific domain-based association tests were performed.

To assess patient characteristics, we compared putative damaging germline *NBN* variant carriers (n=47) or experimentally validated pathogenic and likely pathogenic germline *NBN* variant carriers (n=31) to B-ALL cases with confirmed WT *NBN* status (n=3,719) enrolled in COG AALL0232, P9900, and St. Jude Total 13 and 15 clinical trials. Patient characteristics included age at diagnosis, gender, genetic ancestry, ploidy, fusion genes (*ETV6::RUNX1, BCR::ABL, TCF3::PBX1 and KMT2Arr*), white blood count at diagnosis (WBC), end-of-induction minimal residual disease (MRD) and treatment related events. Fisher’s t-test or non-parametric Wilcoxon rank-sum test was used to assessing statistical significance. Treatment outcome (event-free survival or overall survival) was treated as a time-to-event variable and events included induction failure, relapse, and others (including second malignancies, death, and other events). Its relation to the status of germline *NBN* was assessed by using the Cox Regression with Firth’s Penalized Likelihood model adjusting for age, WBC and study group enrollment. We used R (v4.2.0; The R Foundation, Vienna, Austria) for all statistical analyses, unless otherwise stated.

Further details on experimental procedures and statistical analysis can be found in the **Supplemental Methods**.

## Figures and Tables

**Table 1.** Rare and predicted damaging germline *NBN* variants identified in B-ALL patients (*NBN* NM_002485)

Variant ID	Exon	Class	Genomic Location	Sequence Variant	AF	CADD	REVEL	Designation*	Cases

p.L4P	1	missense	8–90996779-A-G	c.T11C	0	24.6	0.462	VUS	2
p.L18I	2	missense	8–90995069-G-T	c.C52A	1.41E-05	24	0.158	VUS	1
p.Q39K	2	missense	8–90995006-G-T	c.C115A	0	24	0.542	VUS	1
p.S40L	2	missense	8–90995002-G-A	c.C119T	1.19E-05	27	0.88	VUS	2
p.S93L	3	missense	8–90993645-G-A	c.C278T	5.81E-04	25	0.457	VUS**	2
p.M152I	4	missense	8–90992986-C-T	c.G456A	1.13E-04	27.3	0.482	VUS**	7
p.K156N	4	missense	8–90992974-T-G	c.A468C	1.51E-04	26.1	0.438	VUS**	3
p.E179K	5	missense	8–90990497-C-T	c.G535A	3.98E-06	32	0.308	VUS	1
p.V184A	5	missense	8–90990481-A-G	c.T551C	0	25.2	0.179	VUS	1
p.E217Q	6	missense	8–90983454-C-G	c.G649C	7.97E-06	25.1	0.224	VUS	1
p.K219fs	6	frameshift	8–90983441-ATTTGT-A	c.657_661del	2.02E-04	32	NA	Pathogenic	7
p.F222L	6	missense	8–90983439-A-G	c.T664C	1.77E-05	28.2	0.937	VUS	1
p.I228R	6	missense	8–90983420-A-C	c.T683G	7.10E-05	25.7	0.54	VUS	3
p.A241T	7	missense	8–90982767-C-T	c.G721A	1.59E-05	26	0.396	VUS	1
p.F263S	7	missense	8–90982700-A-G	c.T788C	1.95E-04	25.4	0.737	VUS**	2
p.G274R	7	missense	8–90982668-C-T	c.G820A	7.96E-06	24	0.308	VUS	1
p.L281X	7	stop gain	8–90982646-A-C	c.T842G	3.98E-06	33	NA	Pathogenic	1
p.A313V	8	missense	8–90976694-G-A	c.C938T	3.18E-05	26.8	0.661	VUS	1
p.Q448L	10	missense	8–90967565-T-A	c.A1343T	4.25E-05	21.5	0.037	VUS**	1
p.D469Y	11	missense	8–90965912-C-A	c.G1405T	6.16E-05	26.1	0.149	VUS**	1
p.P495L	11	missense	8–90965833-G-A	c.C1484T	3.19E-05	23.9	0.097	VUS	1
p.E552Q	11	missense	8–90965663-C-G	c.G1654C	0	23.6	0.058	VUS	1
p.V556E	11	missense	8–90965650-A-T	c.T1667A	1.99E-05	22.5	0.078	VUS	2
p.E564K	11	missense	8–90965627-C-T	c.G1690A	8.10E-04	22.9	0.081	Benign/Likely benign	5
p.S706X	14	stop gain	8–90955548-G-C	c.C2117G	1.06E-05	42	NA	Pathogenic	1

* NBN variant designation reported in the ClinVar database [https://www.ncbi.nlm.nih.gov/clinvar/?term=nbn%5Bgene%5D&redir=gene, accession date: 03/2023] AF Allele frequency in gnomAD; VUS Variant of uncertain significance

VUS** Variant of uncertain significance with conflicting interpretations of pathogenicity

**Table 2.** B-ALL-related *NBN* variant classification

Variant	Position	Protein Stability^1^	MMC Sensitivity^2^	Final Classification

L4P	4	Complete loss	High	Pathogenic
S40L	40	Complete loss	High	Pathogenic
S706X	706	Complete loss	High	Pathogenic
I228R	228	Complete loss	High	Pathogenic
A313V	313	Complete loss	High	Pathogenic
K219fs	219	Complete loss	High	Pathogenic
L281X	281	Complete loss	High	Pathogenic
F222L	222	Partial loss	Moderate	Likely Pathogenic
M152I	152	Partial loss	WT-like	Likely Pathogenic
S93L	93	Partial loss	Moderate	Likely Pathogenic
K156N	156	Partial loss	Moderate	Likely Pathogenic
E179K	179	Partial loss	WT-like	Likely Pathogenic
V184A	184	Partial loss	WT-like	Likely Pathogenic
Q39K	39	WT-like	Moderate	Likely Pathogenic
F263S	263	Mild loss	WT-like	Likely Benign
E217Q	217	Mild loss	WT-like	Likely Benign
A241T	241	Mild loss	WT-like	Likely Benign
L18I	18	Mild loss	WT-like	Likely Benign
P495L	495	WT-like	WT-like	Likely Benign
E552Q	552	WT-like	WT-like	Likely Benign
G274R	274	WT-like	WT-like	Likely Benign
Q448L	448	WT-like	WT-like	Likely Benign
D469Y	469	WT-like	WT-like	Likely Benign
E564K	564	WT-like	WT-like	Likely Benign
V556E	556	WT-like	WT-like	Likely Benign

1 Variant protein stability comparte to WT NBN stability: *Complete loss* ≥ 90% reduction, *Partial loss* 50–90% reduction, *Mild loss* 20–50% reduction, *WT-like* <20% reduction.

2 MMC sensitivity quantified as fold change in variant frequency pre- and post MMC treatment: *High* <0.75, *Moderate* 0.75–1, *WT-like* >1.

## Data Availability

Sharing of the data collected for the study is not intended. However, we are open to collaborations and requests can be made at any time by e-mail to the corresponding author (jun.yang@stjude.org).
